# Cell-type specific effects of the long non-coding RNA HIF1A-AS3 on HIF1A expression in kidney cells

**DOI:** 10.1038/s41598-025-12441-5

**Published:** 2025-07-30

**Authors:** Simone Reichelt-Wurm, Lena Knauss, Bettina Strasser, Mona Scharf, Kathrin Holler, Elke Eggenhofer, Markus Kretz, Bernhard Banas, Miriam C. Banas

**Affiliations:** 1 Department of Nephrology, University Hospital Regensburg, Franz-Josef-Strauss-Allee 11, 93053 Regensburg, Germany; 2 Department of Surgery, University Hospital Regensburg, Regensburg, Germany; 3 Institute for Molecular Medicine, MSH Medical School Hamburg, Hamburg, Germany

**Keywords:** HIF1A, HIF1A-AS3, HIF1A-AS2, Hypoxia, Hyperglycemia, Oxidative stress, Cell biology, Molecular medicine, Nephrology

## Abstract

The hypoxia-inducible factor 1α (Hif1α) represents the master transcription factor coordinating cellular responses to oxygen depletion. With hundreds of target genes it plays a key role in numerous bio-medical conditions as well as neoplastic and non-cancerous diseases, which in turn requires a strict regulation. Long non-coding RNAs have the potential to virtually control every step of gene expression. We aimed to investigate the expression and role of HIF1A antisense lncRNAs HIF1A-AS1, AS2, and AS3 under hyperglycemic, hypoxic, or both conditions in three non-cancerous human renal cell types: HK-2 cells, primary RPTECs, and mesangial cells. We observed that HIF1A-AS2 and AS3 expression was upregulated under oxygen deprivation. Furthermore, knockdown (KD) of HIF1A-AS3 resulted in a significant reduction of HIF1A-AS2 and even more important of Hif1α in HK-2 cells but not mesangial cells. While KD of HIF1A also had a diminishing effect on HIF1A-AS2 and AS3 RNA levels, KD of HIF1A-AS2 only affected HIF1A-AS3 but not HIF1A. Treating HK-2 cells with Actinomycin D revealed a high HIF1A-AS3 RNA stability. In conclusion, our data reveal a cell-type specific effect of HIF1A-AS3 on HIF1A RNA and protein expression which might allow the development of a cell-type specific HIF1A antagonist based on lncRNAs.

**Supplementary Information:**

The online version contains supplementary material available at 10.1038/s41598-025-12441-5.

## Introduction

The hypoxia-inducible factor 1 (HIF1), consisting of subunit α and β, represents the master transcription factor crucial in orchestrating cellular responses to oxygen (O_2_) deprivation^1^. While rapidly degraded under normoxia (NOX), hypoxia (HOX) leads to Hif1α stabilization facilitating dimerization with Hif1β and translocation in the nucleus^2,3^. Here, Hif1 activates a cascade of adaptive pathways with 314 target genes (according to the Harmonizome 3.0 data set)^4^ to enhance cellular survival and function. Its significance was delineated in various conditions like embryogenesis^5^, transplantation^6,7^, or respiratory diseases as observed recently during the COVID-19 pandemic^8^, but also in malfunction regarding cancer-mediated angiogenesis^9,10^.

In the renal context, Hif1α plays an important role in both acute and chronic kidney disease (CKD) such as ischemia-reperfusion injury (IRI) or diabetic kidney disease (DKD), respectively. IRI significantly impacts renal transplantation outcomes since the temporary interruption as well as the rapid restoration of blood supply cause tissue damage^11,12^. In DKD, hyperglycemia (HG) creates not only a HOX milieu but also represses Hif1α signaling^13–15^.

With its isoform HIF2A, HIF1A shares a common DNA binding site within the hypoxia-response element (HRE). Nevertheless, they exhibit distinct target gene profiles^16^. This, together with its significance for cellular function in HOX, necessitates a strict fine-tuning of Hif1α activity. Long noncoding RNAs (lncRNAs) can achieve this nuanced control of their target genes, including HIF1A. LncRNAs, characterized by a length of at least 200 nucleotides and the lack of protein-coding potential, have emerged as key players in controlling gene expression^17^. Their regulatory influence encompasses pre- and post-transcriptional control, splicing, and genomic imprinting^18,19^, demonstrating significance in cancer^20,21^, allograft rejection^22^, and various diseases such as DKD^23,24^. Intergenic and overlapping lncRNAs, especially in antisense (AS) direction, are assumed to exert a cis-regulatory function on protein-coding genes.

The human HIF1A gene locus, situated on the (+) DNA strand of chromosome 14, harbors three antisense transcripts, named HIF1A-AS1, -AS2, and -AS3. HIF1A-AS1, comprising two exons, overlaps with the first exon of HIF1A and its promoter region, respectively. The single exon lncRNA HIF1A-AS2 is encoded within the last exon of HIF1A and its termination region. According to Reference Sequence (RefSeq) database, the first exon of HIF1A-AS3 localizes approximately 2000 bases upstream of HIF1A-AS2 on the (-) DNA strand while the two remaining exons are found in the first intronic regions of HIF1A.

Several studies address the role of HIF1A-AS1, -AS2, or -AS3 – predominantly in the context of cancer. Here, all are associated with high tumorigenic potential and a poor prognosis for affected patients^20,25,26^. However, others also revealed further roles for HIF1A-AS1 in Coxsackievirus B3 infections^27^, HIF1A-AS2 in hypoxia-induced injury^28^, or HIF1A-AS3 in diabetes^29^.

To our knowledge, the role of HIF1A-AS1, -AS2, and -AS3, in non-cancerous renal cells has not yet been investigated. The aim of this work is to elucidate potential interdependencies between HIF1A/Hif1α and its associated AS lncRNAs as well as to explore possible interactions between individual HIF1A lncRNAa or their combined effects in three renal non-cancerous cell types. LncRNAs, which regulate this transcription factor, are of particular interest, as HIF1A represents a key molecule in various biological processes. The interaction of two or more lncRNAs in controlling their target genes facilitates fine-tuning of regulatory mechanism.

## Results

### HIF1A-AS1, 2, and 3: gene locus, isoforms, conservation, and expression in HK-2 cells

**Fig. 1:**
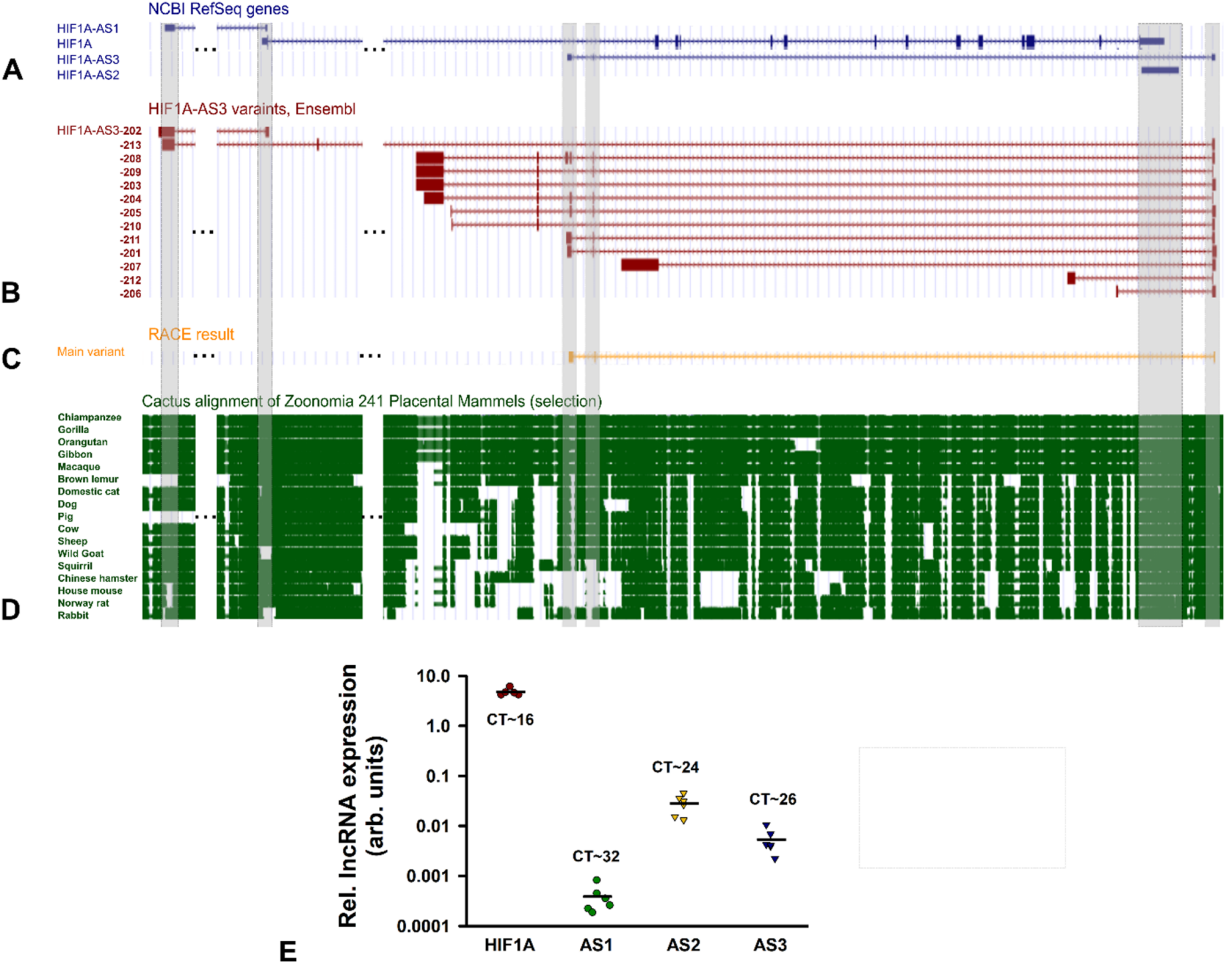
Gene locus of HIF1A mRNA, HIF1A-AS1, AS2, and AS3 lncRNA and their expression in HK-2 cells. (**A**) Modified depiction of the gene locus of HIF1A mRNA, HIF1A-AS1, AS2, and AS3 (all blue) as provided by UCSC genome browser based on current RefSeq database (Annotation Release NCBI RefSeq GCF_000001405.40-RS_2023_10). (**B**) Using the UCSC genome browser tool “costum tracks”, data for HIF1A-AS3 variants from Ensembl genome browser (release 112) are shown in red. (**C**) Illustration of RACE result (yellow) for HIF1A-AS3 sequence analyses was also conducted with “costum tracks” tool. (**D**) A selection of alignments of 17 species (green) compared to human genome assembly is shown, applying the UCSC track Cactus Alignment & Conservation of Zoonomia Placental Mammals (241 Species). Grey bars highlight the exon positions of HIF1A-AS1, AS2, and AS3. For better clarity, genome positions Chr14:61,682,000–61,695,000 and Chr14:61,700,000–61,706,000 were omitted, marked by 3 black dots. (**E**) RNA expression levels of HIF1A, HIF1A-AS1, AS2, or AS3 in untreated HK-2 cells determined by qPCR, depicted as logarithmic plot. Dots and triangles represents single results, bars the mean expression. CT: threshold cycle; *n* = 5–6.

In contrast to RefSeq database (Fig. 1A), the previous (May 2024) Ensembl genome browser release 112 displayed 12 different annotations of HIF1A-AS3 consisting of two to six exons (Fig. 1B). In the meantime, this number increased to 25 transcript variants (Release 113, October 2024). Thus, we utilized rapid amplification of cDNA ends (RACE) and mapped the sequences to the annotated references. Our studies point to only one primarily expressed variant in HK-2 cells: the RefSeq annotated 533 nucleotides-long HIF1A-AS3 version with three exons (RefSeq ID: NR_144368.1), which we focus on in this study (Fig. 1C). Further qPCR examinations also revealed a minor expression of other transcript variants (data not shown).

Using Cactus whole-genome alignment tool which covers 241 species, we ascertained a good or high conservation of HIF1A-AS1 and AS2, respectively (Fig. 1D). In some species, the first or second exon of HIF1A-AS1 is absent, including in mice and rats. The sequence of the first exon of HIF1A-AS3 is well conserved across all species. The second and third exons are well conserved only in higher primates but partially or entirely absent in other species (e.g. mice and rats). Additional alignments, based on Ensembl-annotated exons, further highlight that HIF1A-AS3 is predominantly expressed in higher primates. Notably, some of these exons represent short or long interspersed nuclear elements (SINE or LINE), including Alu sequences which are specific to primates (not shown).

Next, we investigated the RNA expression levels of HIF1A and its AS lncRNAs in HK-2 cells under basal cell culture conditions (Fig. 1E). Showing a mean C_T_ value of 16, a substantial amount of HIF1A mRNA was observed. With average C_T_ values of 24 and 26, respectively, HIF1A-AS2 or AS3 represent moderately expressed genes. Contrarily, HIF1A-AS1 and some Ensembl annotated HIF1A-AS3 variants (data not shown) were very low expressed (C_T_~32 or higher).

### HIF1A-AS2 and AS3 RNA and Hif1α protein are upregulated under hypoxia and hyperglycemia

**Fig. 2:**
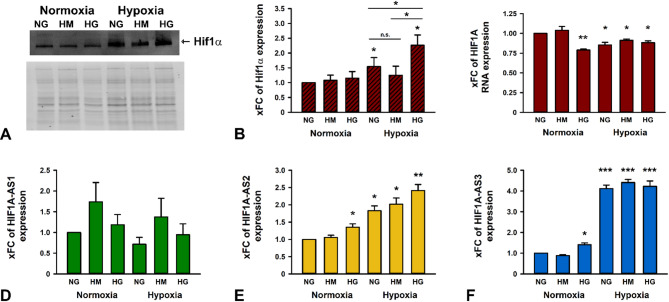
Expression of Hif1α protein, HIF1A mRNA, HIF1A-AS1, AS2, and AS3 lncRNA in HK-2 cells under normal glucose (NG), high mannitol (HM), and high glucose (HG), respectively, under normoxia or hypoxia. (**A**) Detection of Hif1α by western blotting, thereunder whole protein used for normalization. The whole protein stain free western blot image is compressed in length. (**B**) Quantification of Hif1α based on band density and size after total protein normalization. Bars + standard deviation (SD) are shown as x-fold change (xFC) using NG-normoxia samples as reference. (**C**–**F**) RNA expression of HIF1A mRNA, HIF1A-AS1, HIF1A-AS2, and HIF1A-AS3, respectively, shown as xFC with NG-normoxia samples as reference, plotted as bars + SD. *n* = 3–4. Significance was analyzed by ANOVA, followed by Student’s t-test. ^n.s^.not significant; *p < 0.05; **p < 0.001; ***p < 0.00001 compared to NG under normoxia or as indicated otherwise.

Although various factors and circumstances influence the expression and function of Hif1α, our focus is on HG, HOX, or their combination. We observed a significant increase of Hif1α protein expression when HK-2 cells were cultured in a hypoxic chamber. This upregulation was further accentuated in the presence of high glucose concentration suggesting an additive effect (Fig. 2A,B, and Suppl. Figure S1 for whole blot images). Contrarily, applying high mannitol (HM) as osmotic control led to reduced Hif1a expression.

Next, we analyzed RNA expressions and found a slight but significant reduction in HIF1A mRNA levels under both HG and HOX treatments (Fig. 2C). High osmolarity caused a stronger increase of HIF1A-AS1 expression than HG. While HOX alone reduced HIF1A-AS1 level, the addition of either HM or HG had an averting effect. Nevertheless, due to the broad variation in data, these results did not reach statistical significance (Fig. 2D). HG as well as HOX led to a significant increase in HIF1A-AS2 RNA expression with an additive effect when both conditions were combined. Expression changes of HIF1A-AS2 RNA were significant when comparing HM under HOX to basal conditions (NG + NOX), but not when compared to NG or HG under HOX (Fig. 2E). Both HG and HOX, respectively, caused an enhancement in HIF1A-AS3 RNA expression with a modest increase by a factor of 1.6 relative to osmotic control under NOX, or substantially by a factor of 4.1 comparing NOX and HOX treatments. Under O_2_ depletion, neither HM nor HG had a further impacted on HIF1A-AS3 expression (Fig. 2F).

### Bidirectional influence between HIF1A and HIF1A-AS3

Genes sharing a common locus are often co-regulated, however their expressions can either change concordantly or inversely. To reveal the potential for mutual influence in a cis-regulatory manner, we conducted silencing (knockdown, KD) or overexpression (OE) experiments in HK-2 cells and analyzed the impact on the expression of the other RNAs and on Hif1α expression, respectively.

**Fig. 3:**
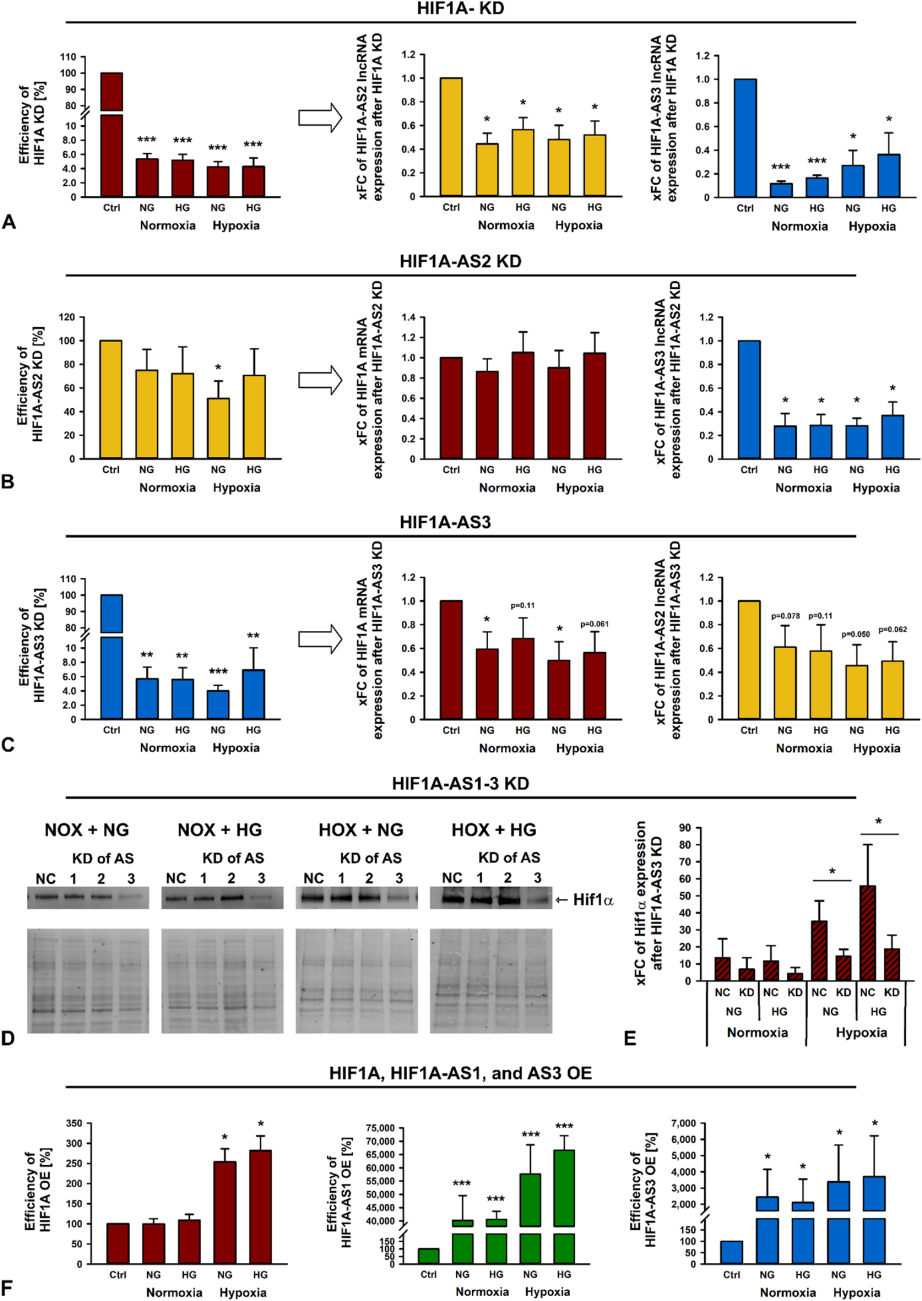
Expression of HIF1A mRNA, HIF1A-AS2, and 3 lncRNA, and Hif1α protein after knockdown (KD) or overexpression (OE) of HIF1A mRNA, HIF1A-AS1, AS2, and AS3 lncRNA, respectively in HK-2 cells under normal glucose (NG) or high glucose (HG), respectively, under normoxia (NOX) or hypoxia (HOX). Efficiency of KD of HIF1A mRNA (**A**), HIF1A-AS2 lncRNA (**B**), and HIF1A-AS3 lncRNA (**C**) and the corresponding effect (indicated by arrow) on HIF1A mRNA and HIF1A-AS2 or AS3 lncRNA expression shown as xfold change (xFC). Data are represented by bars + standard deviation (SD). (**D**) Detection of Hif1α by western blotting after KD of HIF1A-AS1, AS2 and AS3, respectively, under NG or HG and NOX or HOX. Scrambled siPools served as negative control (NC). Thereunder whole protein used for normalization. (**E**) Quantification of Hif1α based on band density and size after total protein normalization after KD of HIF1A-AS3 under NG or HG and NOX or HOX. The whole protein stain free western blot image is compressed in length. Four original immunoblotting images were cropped and merged to with a white space between each blot. Scrambled siPools served as NC. xFCs are represented by bars + SD. (**F**) Efficiency of OE of HIF1A, HIF1A-AS1, and HIF1A-AS3 lncRNA depicted as xFC. Data are represented by bars + SD. *n* = 3–4. Significance was analyzed by ANOVA, followed by Student’s t-test. *p < 0.05; **p < 0.001; ***p < 0.00001 compared to NG + NOX.

First, we conducted silencing of HIF1A mRNA, which was highly efficient and significant under all conditions. This also led to significant decrease of HIF1A-AS2 and even more pronounced of HIF1A-AS3, independent of any other treatment (Fig. 3A). Only under NOX conditions, a KD of HIF1A-AS1 could be achieved. The influence of HIF1A-AS1 silencing on HIF1A, HIF1A-AS2 or AS3 RNA expression was marginal (data not shown). Eventough the combination with other treatments indicated a trend, only silencing of HIF1A-AS2 with coincident HOX (under NG) resulted in a significant reduction of this transcript. An effect on HIF1A expression was not ascertained. In spite of the minor KD efficiency and statistical insignificance, a HIF1A-AS2 KD caused a considerable and significant decline of HIF1A-AS3 RNA under basal, HG, HOX, or both conditions (Fig. 3B). KD of HIF1A-AS3 was highly efficient and significant, independent from any further experimental intervention. Interestingly, a HIF1A-AS3 KD led to a stronger decrease of HIF1A-AS2 RNA than direct silencing of HIF1A-AS2 although the significance threshold was missed narrowly. Moreover, also HIF1A mRNA was ever markedly reduced whereby the 0.05% level of significance was at least closely achieved (Fig. 3C).

LncRNAs can take effect not only on RNA but also on protein level. Thus, we analyzed Hif1α expression via immunoblotting. Applying the same experimental procedure, we found that the negative control (NC) approach provided comparable results to our previous stimulation experiment (Fig. 2). In general, Hif1α expression was low under NOX but strongly increased under HOX and even more pronounced under simultaneous HG (Fig. 3D). Compared to the corresponding NC, significant changes in Hif1α protein expression after KD of HIF1A-AS1 and AS2 were not found. In contrast, silencing HIF1A-AS3 resulted in a clear reduction of Hif1α, which was statistically significant under both HOX conditions (Fig. 3E and Suppl. Figure S2 for whole blot images).

Complementary, OE experiments were conducted. In general, effects were far less pronounced than in KD experiments. Only OEs of HIF1A-AS1 and AS3 were marked and significant in under all conditions. OE of HIF1A mRNA could be induced significantly only under HOX (Fig. 3F), however, a change in Hif1α protein expression after lncRNA OE was not detected (not shown).

In summary, under the conditions examined here, an increase of a particular transcript had only minimal implications for the neighboring genes. In contrast, KD experiments suggest a mutual influence of HIF1A, HIf1A-AS2, and HIf1A-AS3. Additionally, absence of HIf1A-AS3 caused a strong decline of Hif1α protein.

### HIF1A or HIF1A-AS3 KD affect the expression of each other as early as 16 h after Silencing

With the experiments above, we characterized the expression of HIF1A and its adjacent genes 72 h after silencing (24 h KD + 24 h NG/HG + 24 h additional NOX/HOX). Thus, early effects of the KD might not have been revealed. Therefore, we conducted a KD under basal conditions only and harvested the cells every 4–8 h for expression analyses.

**Fig. 4:**
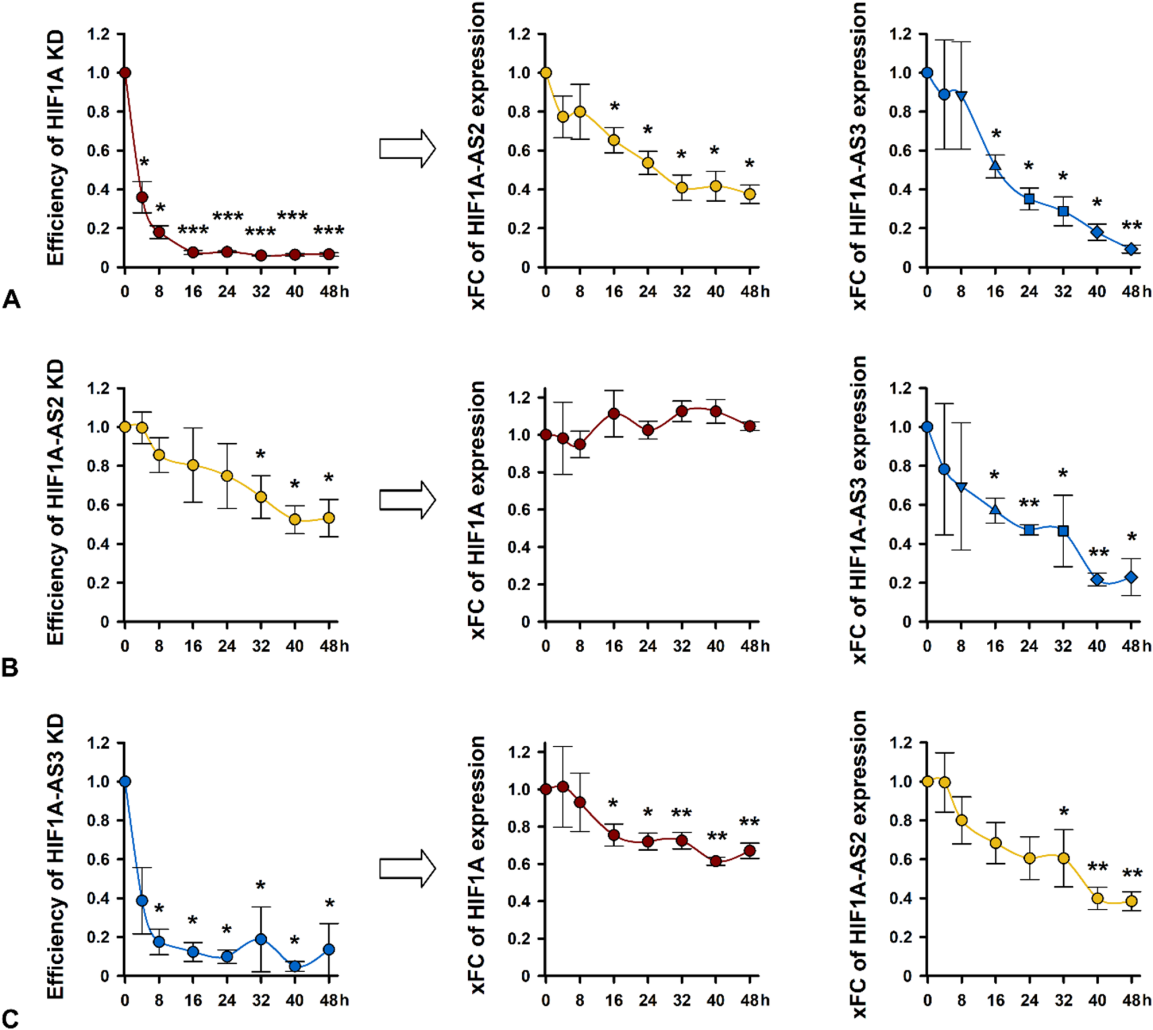
Expression of HIF1A mRNA, HIF1A-AS2, and AS3 lncRNA, after knockdown (KD) of HIF1A mRNA, HIF1A-AS1, AS2, and AS3 lncRNA, respectively in HK-2 cells under basal conditions. Efficiency of KD of HIF1A mRNA (**A**), HIF1A-AS2 lncRNA (**B**), and HIF1A-AS3 lncRNA (**C**) and the corresponding effect (indicated by arrow) on HIF1A mRNA and HIF1A-AS2 or AS3 lncRNA expression shown as xfold change (xFC). Data are represented by bars + standard deviation (SD) compared to scrambled siPools treated HK-2 cells which served as NC. *n* = 4. Significance was analyzed by ANOVA, followed by Student’s t-test. *p < 0.05; **p < 0.001; ***p < 0.00001 compared to NC.

Silencing of HIF1A reduced the amount of HIF1A RNA to 36% within only 4 h and to considerably less than 10% after 16 h. From that moment on, a distinct decrease of HIF1A-AS2 and even more of HIF1A-AS3 was observable (Fig. 4A). Silencing of HIF1A-AS2 resulted in a halving of HIF1A-AS2 RNA within 40 h. Interestingly, after 16 h the HIF1A-AS2 expression was reduced only by 20% but HIF1A-AS3 RNA declined by 44%. There was no impact on HIF1A mRNA expression. This is noteworthy since the strong decrease of HIF1A-AS3 did not lead to a time-delayed diminishment of HIF1A mRNA (Fig. 4B). HIF1A-AS3 KD exerted its effect already within 4 h, the expression remained very low over the entire observation period. HIF1A mRNA and HIF1A-AS2 lncRNA were also significantly reduced after 16–32 h (Fig. 4C).

Recapitulating, our analyses revealed that HIF1A und HIF1A-AS3 affected each other and the expression of HIF1A-AS2 while HIF1A-AS2 silencing caused only a reduction of HIF1A-AS3 lncRNA but not HIF1A mRNA.

### Incomplete rescue suggests non-reciprocal control of HIF1A and its LncRNAs

**Fig. 5:**
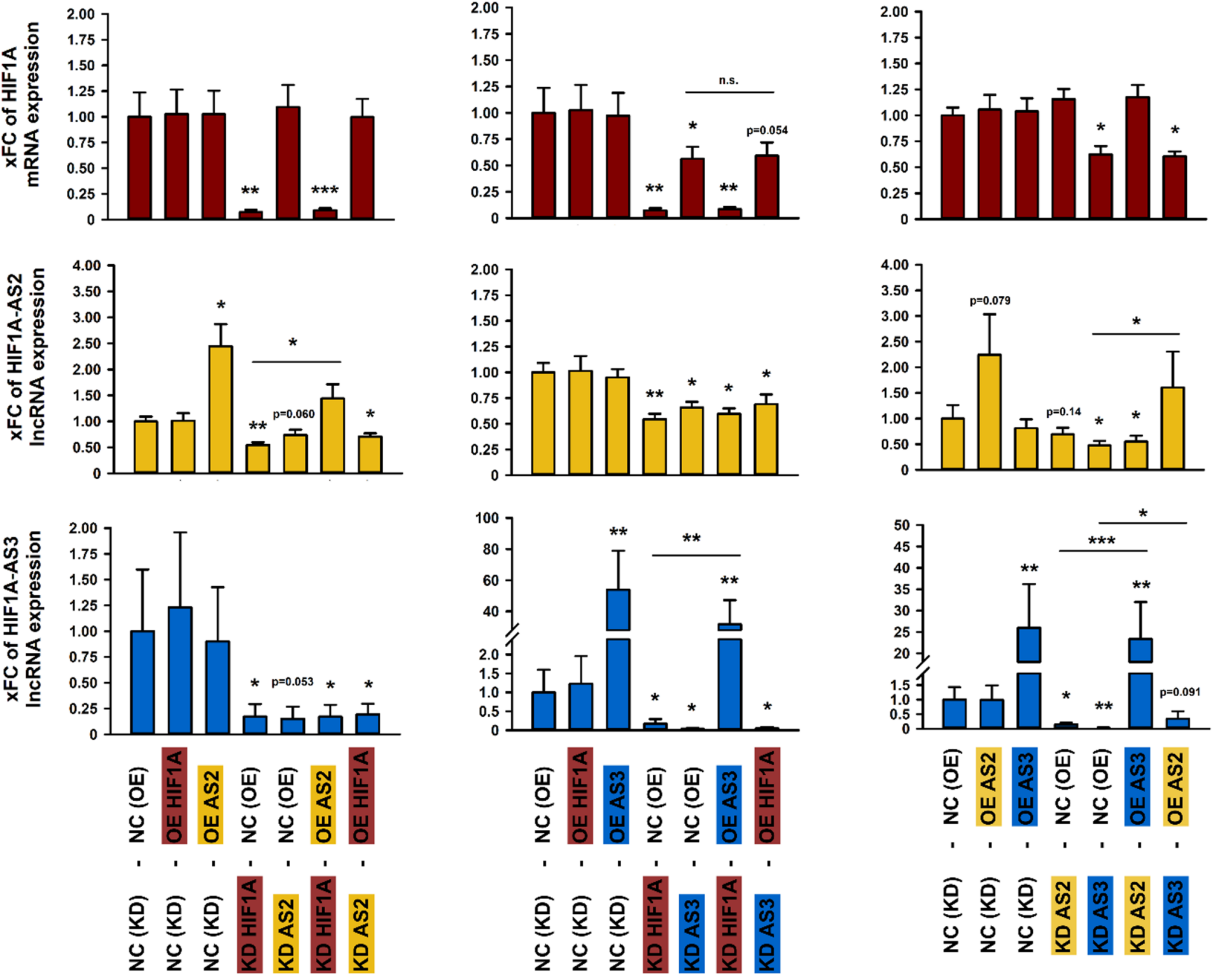
Rescue assays to characterize potential mutual dependencies in expression of HIF1A, HIF1A-AS2 or AS3 in HK-2 cells under basal conditions. The upper panel (red bars) shows x-fold change (xFC) of HIF1A, the second (yellow bars) or third panel (blue bars) display xFC of HIF1A-AS2 and AS3, respectively. As depicted below the third panel, experiments were conducted with “NC (KD)” as knockdown (KD) with scrambled siPools as negative control (NC) and “KD HIF1A”, “KD HIF1A-AS2”, or “KD HIF1A-AS3” as KD for the corresponding RNA. After each KD, an overexpression (OE) experiment was performed using either an empty pcDNA vector as NC “NC (OE)” or a pcDNA carrying the sequence of HIF1A, HIF1A-AS2 or AS3: “OE HIF1A”, “OE HIF1A-AS2”, or “OE HIF1A-AS3”. *n* = 4. Significance was analyzed by ANOVA, followed by Student’s t-test. *p < 0.05; **p < 0.001; ***p < 0.00001 compared to NC or as otherwise specified.

Rescue assays were performed to further elucidate potential mutual dependencies in expression. Thus, we conducted combined KD and OE experiments of HIF1A, HIF1A-AS2 or AS3. We observed a significant reduction of HIF1A mRNA after HIF1A-AS3 was silenced. This effect could not be abolished by OE of HIF1A. Silencing of HIF1A or HIF1A-AS3 KD resulted in a strong and statistically significant reduction of HIFA-AS2. However, HIF1A OE could not reverse the effect of HIF1A-AS3 KD and vice versa. Only subsequent OE of HIFA-AS2 rescued HIFA-AS2 although expression remained below the level of control cells. Finally, silencing of HIF1A-AS3 was achieved by KD of HIF1A, HIF1A-AS2 or AS3. OE of HIF1A-AS3 abolished these effects. Interestingly, HIF1-AS2 OE after HIF1A-AS3 KD increased HIF1-AS3 expression by a factor of approximately 13, although HIF1-AS2 OE alone had no effect on HIF1-AS3 expression. Noteworthily, HIF1-AS2 OE was not sufficient to entirely overcome HIF1-AS3 silencing (Fig. 5).

### HIF1A-AS3 exhibits high RNA stability

To further characterize HIF1A and its neighboring AS lncRNAs, we aimed to conduct RNA stability assays using Actinomycin D (ActD), an inhibitor of RNA synthesis, under NOX and HOX. Because of the experimental setup, HOX should be induced by CoCl_2_ instead of a hypoxic chamber. Therefore, we first validated whether both methods delivered comparable results (Suppl. Figure S3). HIF1A and HFI1A-AS3 were successfully silenced under both conditions (24 h HOX). Moreover, the effect on the expression of the other as well as on HIF1A-AS2 was almost identical to the results shown in Fig. 4 regardless of the method used to induce HOX.

**Fig. 6:**
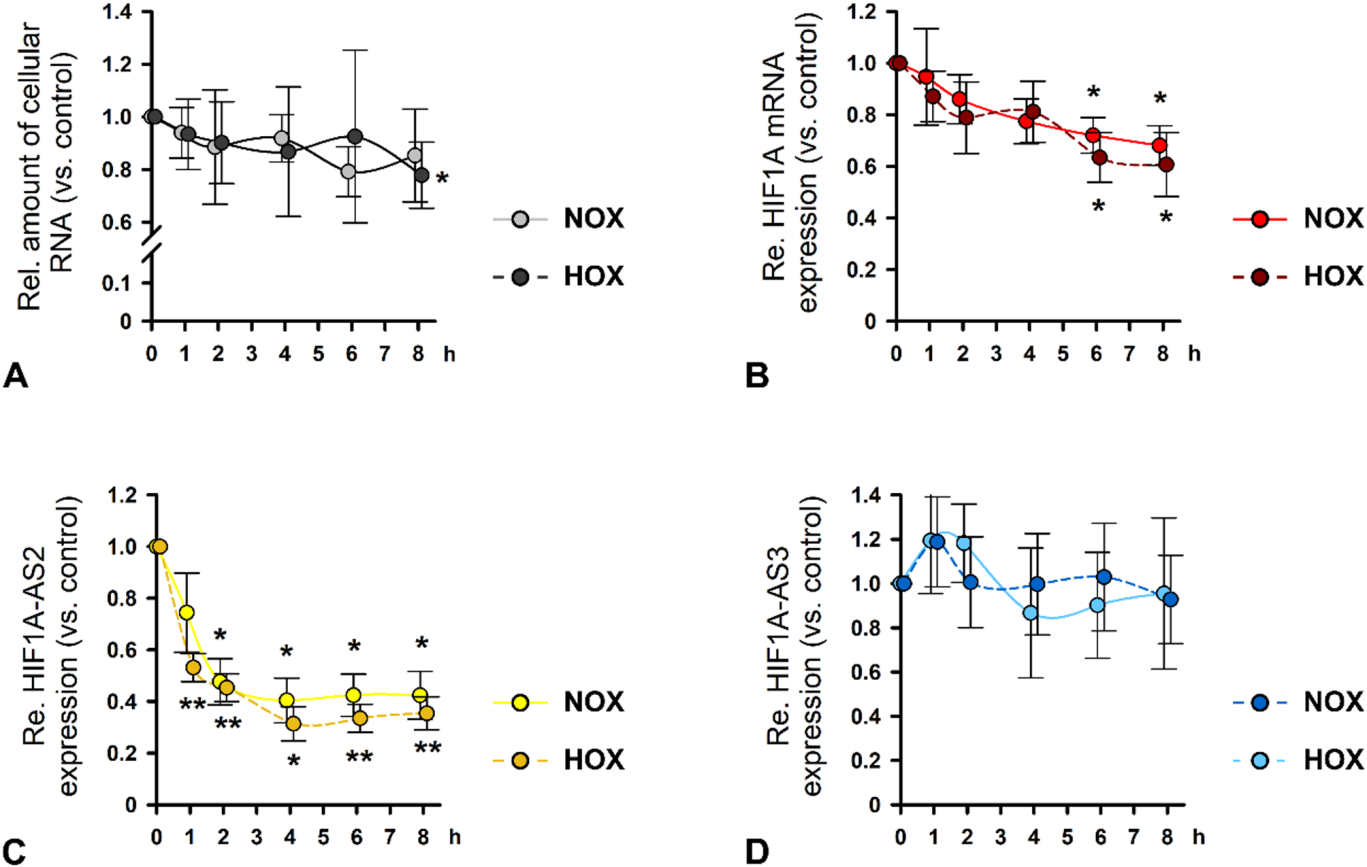
RNA stability assay in HK-2 cells under normoxia (NOX) and hypoxia (HOX). Cells were treated with actinomnycin D (ActD) or DMSO as control for 8 h. (**A**) Amount of whole RNA isolated from HK-2 cells under NOX (light grey) or HOX (dark grey) and simultaneous exposure to ActD relatively to DMSO-treated cells. Relative expression of HIF1A mRNA (**B**), HIF1A-AS2 lncRNA (**C**), or HIF1A-AS3 lncRNA (**D**) in ActD-treated vs. DMSO-treated cells was determined by qPCR. The experiments were conducted under NOX indicated by light red, light yellow, and, light blue, respectively, or under HOX indicated by dark red, dark yellow, and, dark blue, respectively. Error bars represent standard deviation. *n* = 3–4. Significance was analyzed by ANOVA, followed by Student’s t-test. *p < 0.05; **p < 0.001 compared to corresponding DMSO control.

Compared to DMSO treated control cells, the amount of whole RNA remained very stable over the entire time of observation. Solely RNA amount 8 h after application of ActD and under HOX was significantly less than in the control sample (Fig. 6A). HIF1A mRNA levels decreased slowly but significantly after ActD was added (Fig. 6B). Cellular HFI1A-AS2 expression markedly declined after 2 h reaching a stable plateau at 30–40% compared to base level, independent from O_2_ concentration (Fig. 6C). Contrarily, amount of HFI1A-AS3 RNA did not exhibit any signs of decay either under NOX or HOX (Fig. 6D).

### HIF1A-AS3 does not affect Hif1α protein expression in human mesangial cells

Finally, we aimed to analyze other renal cell types to provide a deeper insight into putative cell-specific expression and function of HIF1A AS lncRNAs. First, we decided to investigate RNA expression in primary human renal proximal tubule epithelial cells (RPTECs). Contrarily to HK-2, which were also isolated from the proximal tubule, RPTECs were not immortalized. Given this similarity, we anticipated comparable results.

The expression of HIF1A and HIF1A-AS1 was slightly lower in RPTECs compared to HK-2 cells while HFI1A-AS2 exhibited a moderately higher expression. In contrast, the expression level of HIF1A-AS3 was exceedingly low (Suppl. Figure S4A). Next, RPTECs were subjected to the same experimental setup as HK-2 cells. For HIF1A and HIF1A-AS1, we found no relevant differences in RNA expression profiles compared to those observed in HK-2 cells. An elevated but not significant HFI1A-AS2 expression under HOX was measured. Contrarily, while HIF1A-AS3 under NOX and HG was even below the detection limit, HOX resulted in a pronounced increase of expression. An additive effect of HG was not ascertained (Suppl. Figure S4B-E). Interestingly, the here determined C_T_ value of approximately 26, reached a level compared to this assessed in HK-2 cells (data not shown). Nevertheless, subsequent KD experiments were not feasible since HIF1A-AS3 basal expression was too low.

Compared to hypoxia, our results revealed a minor role of hyperglycemia regarding a potential effect on the expression of HIF1A and its AS lncRNAs in HK-2 cells. Considering also the fact that lncRNAs often exhibit a cell type specific expression, we aimed to analyze an additional cell line in terms of its expression profile. We chose human mesangial cells (HMCs) since others reported contradictory regulation of HIF1A in HK-2 cells and hMCs^30–32^. Thus, we repeated stimulation, KD, and OE experiments in human mesangial cells (HMCs).

**Fig. 7:**
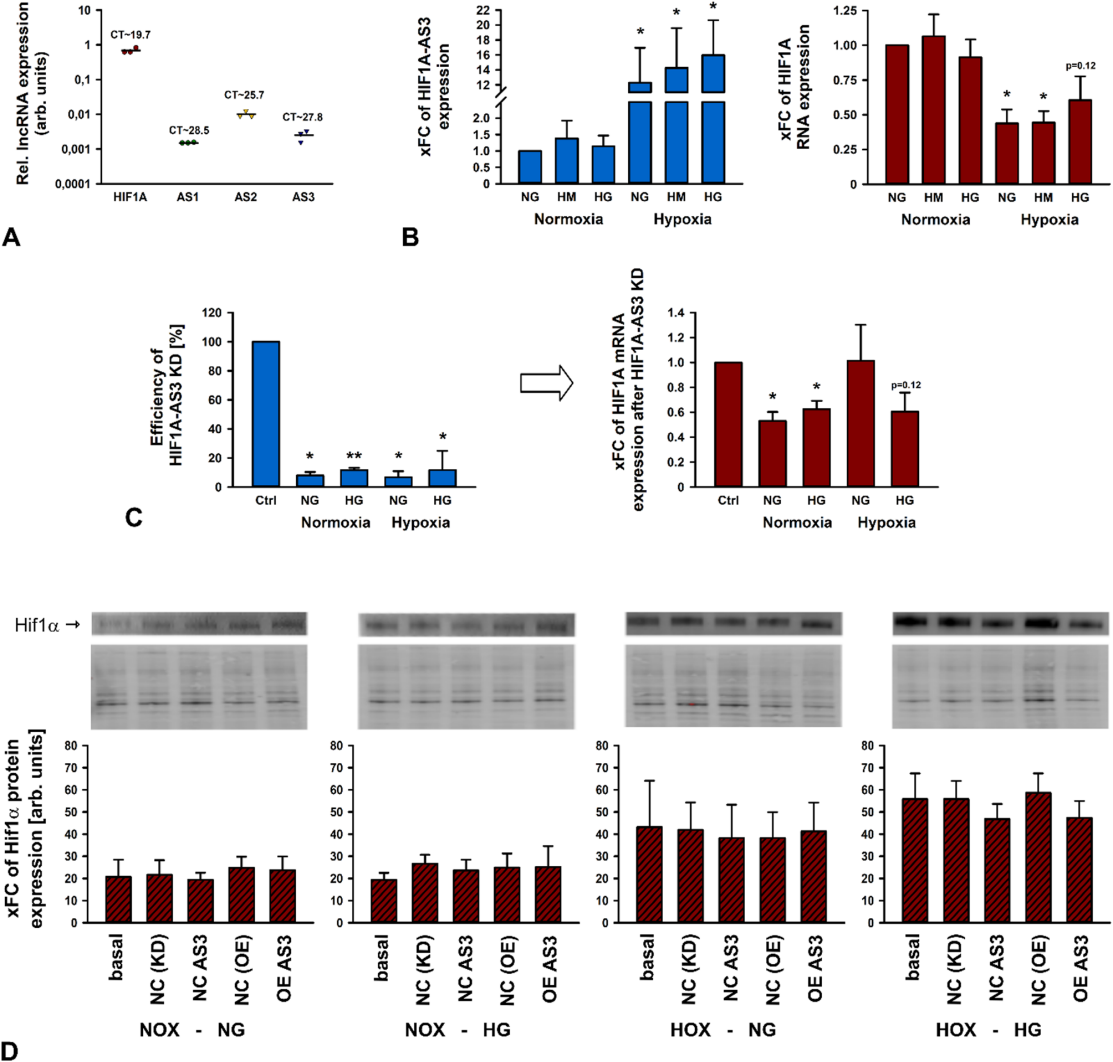
Expression of Hif1α protein, HIF1A mRNA, HIF1A-AS1, and AS3 lncRNA in hMCs after stimulation with high mannitol (HM), and high glucose (HG), respectively, under normoxia (NOX) or hypoxia (HOX) as well as knockdown (KD) and overexpression (OE) experiments. (**A**) RNA expression levels of HIF1A, HIF1A-AS1, AS2, or AS3 in untreated hMCs determined by qPCR, depicted as logarithmic plot. Dots and triangles represents single results, bars the mean expression. C_T_: threshold cycle. (**B**) X-fold changes (xFC) of HIF1A (blue) and HIF1A-AS3 (red) RNA expression under normal glucose (NG), high mannitol (HM) as osmotic control, and high glucose (HG), respectively. Cells were simultansouly exposed to normoxia or hypoxia. Data are represnted by bars + standard deviation (SD). (**C**) OE efficiency of HHIF1A-AS3 lncRNA and the corresponding effect (indicated by arrow) on HIF1A mRNA expression shown as xFC with bars + SD. (**D**) Detection of Hif1α by western blotting after OE of HIF1A-AS3 under NG or HG and NOX or HOX. Scrambled siPools or empty vector served as negative control (both NC); basal: untreated cells. Thereunder whole protein used for normalization and quantification of Hif1α based on band density and size after total protein normalization shown as xFCs, The whole protein stain free western blot image is compressed in length. Four original immunoblotting images were cropped and merged to with a white space between each blot. xFCs are represented by bars + SD represented by bars + SD. *n* = 3 Significance was analyzed by ANOVA, followed by Student’s t-test. *p < 0.05; **p < 0.001 compared to NG.

Here, we ascertained a slightly lower expression of HIF1A-AS3 (and HIF1A-AS2) compared to HK-2 cells while HIF1A-AS1 expression was clearly elevated. C_T_ value of HIF1A was 4 cycles lower (Fig. 7A). Like with HK-2 cells, HOX caused a significant upregulation of HIF1A-AS3, whereas an additive effect of HG was also not observed. Concurrently, a significant reduction of HIF1A mRNA was detected (Fig. 7B). Our analyses could not reveal significant expression changes for HIF1A-AS1 and AS2 (data not shown). Next, HIF1A-AS3 KD and OE experiments were conducted. Silencing HIF1A-AS3 resulted in a highly efficient reduction of this transcript. Like with HK-2 cells, the expression of HIF1A mRNA markedly declined as consequence of reduced levels of HIF1A-AS3 (Fig. 7C). OE expression of HIF1A-AS3 did not lead to significant changes in HIF1A-AS3 or HIF1A RNA expression (not shown).

Furthermore, we analyzed Hif1α protein expression after KD and OE of HIF1A-AS3, respectively, under NOX or HOX and NG or HG. Exposing HMCs to HG was not relevant for Hif1α expression, only HOX caused an increase of detected protein. Surprisingly and in contrast to HK-2 cells, where silencing of HIF1A-AS3 strongly affected Hif1α expression, Hif1α expression remained unchanged. Furthermore, OE of HIF1A-AS3 also showed no effect on protein level (Fig. 7D and Suppl. Figure S5 for whole blot images).

## Discussion

In recent years, lncRNAs received raising attention since they have the potential to virtually control every step of gene expression. Thus, they represent conceivable objectives for new therapies. Manipulating a regulatory lncRNA can directly affect its target gene. With this study, we addressed HIF1A AS lncRNAs, especially HIF1A-AS3, as putative regulators of HIF1A/Hif1α and each other in a mutual manner.

Hif1α is characterized as master transcription factor being upregulated under O_2_ deprivation^1^. Compared to other organs, kidneys are inherently more susceptible to hypoxia^33^. However, in the diabetic kidney, HG is considered to further promote the development of a hypoxic milieu. As reviewed by others, increased oxygen consumption is a consequence of glomerular hyperfiltration and higher transmembrane transporter activity. Contrary to the obvious assumption, HG does not lead inevitably to enhanced Hif1α activity since elevated glucose levels rather facilitate PHD-mediated Hif1α degradation^13–15^, .

PHDs serve as main regulators of Hif1α protein expression^2,3^. Our western blotting analysis revealed an increase of Hif1α under HOX with NG and even more under HOX plus HG in HK-2 cells. For the latter, HM had no or rather an averting effect. Under NOX, neither HM nor HG affected Hif1α protein expression. The classification of these results is challenging. Owczarek et al. reported significantly higher Hif1α protein level under NOX and HG. As expected, HOX resulted in an increase in Hif1α, but additional treatment of HG had no further impact^34^. In contrast, García-Pastor et al. demonstrated a steady decrease in Hif1α when HK-2 cells were subjected to HG and HOX over a 16-hour period^31^. We think that basal media composition plays a key role. García-Pastor et al. used DMEM/F12 medium, which contains 3151 mg/L glucose^31^. We and others^34^ used Keratinocyte serum-free medium. The media composition is not released by the manufacturer but upon request we received the information that the glucose concentration of this medium is approximately 1000 mg/L. This represents a physiological glucose level while employing DMEM/F12 medium could lead to a permanent pre-stimulation of HK-2 cells causing different results.

In contrast to HIF1A-AS1, we also observed a HOX or HG and HOX mediated increase of HFI1A-AS3 and AS2 lncRNA expression, respectively, in HK-2 cells. Repeating this experiment with RPTECs provided results with close resemblance. Since HK-2 cells and RPTECs exhibited for HIF1A-AS3 a C_T_ value of approximately 26 after induction of HOX, we speculate that this transcript might function in the same way in both cell types although it is considerably less expressed in RPTECs under basal conditions. Further investigations are necessary here.

To our knowledge, the influence of HG on HIF1A-AS1, AS2, or AS3 is barely investigated, especially in non-cancerous cells. Others reported an elevated HIF1A-AS2 expression in patients with diabetic retinopathy^35^, but a decreased HIF1A-AS3 expression in diabetic patients suffering from peripheral arterial disease^29^. Hypoxia caused an increased expression of all three lncRNAs, at least in cancer cells^36–38^ but also in endothelial cells regarding HIF1A-AS2^39^.

Our KD experiments and rescue assays suggest that HIF1A-AS3 strongly affects the expression of HIF1A and HIF1A-AS2. For both genes, we also ascertained a mutual effect regarding HIF1A-AS3 expression. Interestingly, efficiency of HIF1A-AS2 KD itself was inferior to its effect on HIF1A-AS3 expression. Furthermore, there was no influence on HIF1A expression. This is surprising, considering that KD of HIF1A-AS2 led to a fast and strong decline of HIF1A-AS3 but this reductions did not affect HFI1A expression. It seems that direct silencing of HIF1A-AS3 downregulates HIF1A mRNA but not indirect silencing via HIF1A-AS2 KD. The underlying molecular mechanisms remains to be elucidated. This also applies to the mutual dependency of HIF1A-AS2 and AS3, as revealed by our rescue assays where the KD of one can be abolished by OE of the other (at least partially). Nevertheless, the biological function might be relevant since an elevated HIF1A-AS2 expression was observed in various cancers^20,40,41^, especially in the advanced stage of tumor progression^42^. An upregulation of this lncRNA was associated with increased proliferation, migration, and invasion of tumor cells and a poor patient survival rate^43,44^. Maybe the fact, that HIF1A-AS2 and AS3 are encoded in spatial proximity plays a role: the promoter region of HIF1A-AS2 overlaps with the first intron of HIF1A-AS3.

Although Hif1α is mainly controlled by PHDs on protein level^2,3^, El-Naggar et al. identified Y-box binding protein 1 (YBX1) as regulator of HIF1A mRNA expression^45^. Recently, others reported an interaction of HIF1A-AS3 with YBX1 determining a positive effect on the Hif1α mediated transcription activation^26,46^. This might explain the reduced HIF1A mRNA expression after silencing of HIF1A-AS3. However, further investigations are necessary to elucidate the underlying mechanism.

Our analyses revealed that only the KD of HIF1A-AS3 caused a significant reduction of Hif1α protein in HK-2 cells but not in hMCs. The control mechanisms of Hif1α vary between different cell types. Exposing murine and human mesangial cells to HG resulted in an upregulation of Hif1α – in contrast to human RPTECs^30^. This increase occurs in a carbohydrate-responsive element-binding protein (ChREBP)-dependent manner^47,48^. Contrarily, we observed a decreased HIF1A mRNA expression under HOX with NG or HM in hMCs. Combining HOX with HG alleviated this effect. Under HOX, Hif1α protein expression increased, additional exposure to HG magnified this effect. Surprisingly, KD of HIF1A-AS3 did not lead to diminished Hif1α protein amounts, regardless of the treatment. It might be possible that lncRNAs do not only display cell-type specific expression patterns but also cell-type specific functions. Otherwise, an unknown factor may protect Hif1α from degradation when HIF1A-AS3 is absent.

Beside its role under physiologic conditions, Hif1α plays a crucial in various cancer. It is associated with the “Warburg effect”^49^, which describes the reprogramming of glucose metabolism in neoplastic cells. As reviewed by others, Hif1α causes an upregulation of genes involved in glucose transport and enzymatic processing^50–52^. In this context, Zheng et al. demonstrated that HIF1A-AS3, the authors called it “HIFAL” (HIF1α anti-sense lncRNA) promotes the association of s prolyl hydroxylase 3 (PHD3) to pyruvate kinase which represents an important step before transcriptional activation by Hif1α, which promotes glycolysis in several breast cancer cell lines^38^.

Considering our results and the results of others^26,38,46^, HIF1A-AS3 seems to play a key part in regulating HIF1 mRNA and Hif1α protein expression as well as Hif1α mediated expression, which makes it an attractive target for nucleic acid therapeutics (NATs). NATs might allow a highly specific but also widespread manipulation of molecular mechanisms. However, further investigations are necessary to analyze the role of HIF1A-AS3 in detail. This also applies to its high stability as shown by our ActD assay. While the concentration of HIF1A-AS2 decreased fast within two hours, HIF1A mRNA showed only a very low reduction over the time - a characteristic which was already demonstrated by others^53^. HIF1A-AS3 remained stable, even under HOX. This indicates that HIF1A-AS3 has a low turnover, and its cellular concentration is maintained by high stability, but not continuous expression. The reason for this high stability needs to be elucidated, maybe HIF1A-AS3 is protected from degradation by its structure or folding or by nucleotide modifications. Circumstances like this high RNA stability have to be considered when HIF1A-AS3 shall serve as target of NATs.

In summary, our data revealed for the first time the expression of HIF1A-AS3 in human renal non-cancer cell lines: HK-2, RPTECs, and hMCs. We showed differences in expression levels and demonstrated that HIF1A-AS3 affects the expression of HIF1A-AS2 and HIF1A mRNA as well as Hif1α protein in HK-2 cells but not in hMCs. However, more tissues and cell types – neoplastic and non-cancerous – have to be analyzed to fully understand the underlying molecular mechanisms of how HIF1A-AS3 affects Hif1α and the expression of other lncRNAs. The regulation of Hif1α is highly crucial since this molecule has key functions in various medical circumstances such transplantation and diseases, especially in diabetes, tumor biology, and tumor angiogenesis.

## Methods

### Cell culture maintenance

In-vitro experiments were conducted with immortalized HK-2 (American Type Culture Collection (ATCC), Manassas, VA, USA), RPTECs (Innoprot, Derio, Spain) and hMCs (Clonetics Corp.; San Diego, CA, USA), respectively. The latter was maintained and characterized as described previously^54^. All cell lines were cultured under sterile conditions at 37 °C in an atmosphere of 5% CO2/95% air (NOX). For HK-2 medium preparation, we used Keratinocyte-SF Medium Kit. HMC were cultured in Dulbecco’s modified Eagle’s medium (DMEM 21855; ThermoFisher Scientific Inc., Waltham, MA, USA) supplemented with 10% fetal bovine serum (FBS; Sigma Aldrich, Taufkirchen. Germany). To both media, 100 U/ml penicillin and 100 µg/mL streptomycin (P/S) (ThermoFisher Scientific Inc., Waltham, MA, USA) were added. All components of RPTEC culture medium were purchased from Innoprot (Derio, Spain) and mixed as follows: 500 ml RPTEC Basal Medium, 25 ml FBS, 5 ml of Endothelial Cell Growth Supplement (ECGS), and 5 ml of P/S solution.

### Induction of HM, HG, or HOX

HK-2, RPTECs, or hMCs were seeded at 200.000 cells per well in 6-well plates and allowed to settle overnight. Medium was replaced for new containing either no further additives (NG), 25 mM mannitol (HM; additionally to ca. 5.0 mM glucose in both media) as osmotic control, or glucose ad 30 mM (HG). Cells were kept under these conditions for 24 h before they were exposed to HOX (or still NOX as control) maintaining the particular medium conditions. HOX was achieved by passing with gas containing 95% N_2_, 5% CO_2_ (Linde GmbH, Unterschleißheim, Germany) in a Modular Incubator Chamber (MIC; Embrient Inc, San Diego, CA, USA). After 24 h cells were harvested for RNA or protein extraction using the NucleoSpin TriPrep Kit (Macherey-Nagel GmbH, Düren, Deutschland). For experiments with additional KD or OE, cells were transfected instead for 24 h as described in the following section.

### Knockdown experiments under NOX/HOX and NG/HG and analysis of early Silencing effects

KD was achieved with siPools (siTOOLs Biotech GmbH, Planegg, Germany) targeting HIF1A, HIF1A-AS1, AS2, or AS3 RNA; scrambled RNA siPOOLs served as NC. One transfection complex mixture contained 492 µl Opti-MEM with 4 µl Lipofectamin RNAiMAX (both ThermoFisher Scientific Inc., Waltham, MA, USA) and 4 µl of the corresponding siPool reaching a final concentration of 6.67 nM. After an incubation time of 15 min and before applying it to the cells, 1.5 ml of FCS- and P/S-free medium was added to the transfection mixture. After 24 h cells were treated with NG or HG for 24 h and for further 24 h additionally with NOX or HOX as delineated above prior to lysing employing the NucleoSpin TriPrep Kit for RNA and protein isolation.

The analysis of early silencing effects was done under NOX and NG conditions only. The KD transfection mixture was prepared as described above and added to 200.000 HK-2 cells per well (6-well plates). Cells were grown for 4 h, 8 h, 16 h, 24 h, 32 h, 40 h, and 48 h and harvested for RNA expression analysis using the NucleoSpin RNA Plus Kit (Macherey-Nagel GmbH, Düren, Deutschland).

### Overexpression experiments

For one well (in a 6 well-plate) an OE transfection mixture prepared composed of 1 µg of the corresponding pcDNA3.1(+) expression vector encoding HIF1A, HIF1A-AS1, AS2, or AS3 as well as 4.0 µl RotiFect Plus (Carl Roth GmbH, Karlsruhe, Germany), and Opti MEM medium to a final volume of 50 µl. An empty pcDNA3.1(+) vector served as NC. The transfection complex was formed within 20 min and then added to the cells in 950 µl culture medium for 24 h. Like with KD experiments, cells received the identical further processing with NG or HG and NOX or HOX for the same period of time before cells were harvested by means of the NucleoSpin TriPrep Kit.

### Rescue assays

HK-2 cells were transfected in solution overnight for 24 h with the transfection mixture described above using siPools targeting HIF1A, HIF1A-AS2, AS3, or scrambled RNA. Then cells received fresh medium and an OE transfections mixture with pcDNA3.1(+) vector encoding HIF1A, HIF1A-AS2, or AS3 as well as an empty vector as NC. After 24 h, we replaced the old medium with fresh medium and incubated the cells for another 24 h before performing RNA isolation using the NucleoSpin RNA Plus Kit.

### Actinomycin D treatment

We subcultured 200.000 HK-2 cells per well in 6-well plates and allowed to adhere over night for 18 h. Cells were exposed to 1 µg/ml Actinomycin D (ActD; Carl Roth GmbH, Karlsruhe, Germany) or dimethyl sulfoxide (DMSO) in the control approach. Simultaneously, cells were kept under NOX or HOX applying 200 µM CoCl_2_ before lysis was conducted after 1 h, 2 h, 4 h, 6 h, and 8 h using the NucleoSpin RNA Plus Kit.

### RNA extraction and expression analysis

The NucleoSpin TriPrep Kit was employed to obtain RNA and protein from the same sample. Extraction was conducted according to manufacturer’s instructions. For RNA extraction only, we utilized the NucleoSpin RNA Plus Kit. In both cases, an on-column DNase I (Qiagen, Hilden, Germany) treatment was performed. Prior to cDNA synthesis using the MMLV reverse transcriptase system, including random primers and RNasin (all Promega, Fitchburg, Wisconsin, USA), RNA concentration was determined by means of the NanoDrop 2000c spectrophotometer (PEQLAB Biotechnologie GmbH, Erlangen, Germany). Subsequent, qPCR experiments were performed applying the QuantiTect SYBR Green PCR kit (Qiagen, Hilden, Germany). All samples were run in triplicates employing the ViiA 7 Real-Time PCR System (ThermoFisher Scientific Inc., Waltham, MA, USA). The xFC was calculated based on the 2^−ΔΔCT^ method with peptidylprolyl isomerase B as reference gene. Primers were designed using Primer3 software and sequences are shown in Table 1.

**Table 1:** Primer sequences.

	Sequence
Primers for qPCR and sequencing	
hHIF1a_for	AGTTCGCATCTTGATAAGGCC
hHIF1a_rev	TCATCTGTGCTTTCATGTCATCT
hHIF1A_AS1_for	TCCACACGCGGAGAAGAG
hHIF1A_AS1_rev	AGGCAGAGACGAGATGAACA
hHIF1A_AS2_for	GTAAAGGACCTAAGGCTCTGG
hHIF1A_AS2_rev	TCACTATGAATCCCTGCACCT
hHIF1A_AS3_for	TGGGCAGCGTCTTGGAAAA
hHIF1A_AS3_rev	CAAGGCAGAAGGATCGCTTT
hCyclo_for	TGTGGTGTTTGGCAAAGTTC
hCyclo_rev	GCTTCTCCACCTCGATCTTG
pCRII-TOPO_for	TGACCATGATTACGCCAAGC
pCRII-TOPO_rev	CACTATAGGGCGAATTGGGC
pMiniT2.0_for	CTGCAGGAAGGTTTAAACGCA
pMiniT2.0_rev	GCCCGCGAAATTAATACGACT
pcDNA3.1_for	CAGTACATCAATGGGCGTGG
pcDNA3.1_rev	TTGTCTTCCCAATCCTCCCC
Primers for cloning	
Clon_hHIF1a_for	GGTACCGATTCACCATGGAGGGC
Clon_hHIF1a_rev	TCTAGACTGCTTTAGGTAATGAGCCACC
Clon_hHIF1a_AS1_for	AAGCTTCGCCGCCGGCGCCCTCCAT
Clon_hHIF1a_AS1_rev	TCTAGATAATGGAACATTTCTTCTCCC
Clon_hHIF1a_AS2_for	GGTACCTGTCCTCCATTGTAAGATAA
Clon_hHIF1a_AS2_rev	GGATCCTACTGCAGGGTGAAGAATTA
Clon_hHIF1a_AS3_for	AAGCTTTCATGTAGCGCCAGCCACAC
Clon_hHIF1a_AS3_rev	TCTAGATATTAACGGAGTAATGTTGGG
Primers and oligos for 5‘ and 3‘ RACE	
5‘-TSO	AAGCAGTGGTATCAACGCAGAGTACATaagacgtggtatcaacgcagtacatrGrGrG
3’ oligo dT primer	CGCAGAGTACATaagacgtggtatcaacgcagtacatTTTTTTTTTTTTTTTTTTTTVN
TSO_outer	GCAGTGGTATCAACGCAGAGT
TSO_inner	AAGACGTGGTATCAACGCAG
HIF1A-AS3_RACE_1	TTGAGAATTCTTCCAGGGGAC
HIF1A-AS3_RACE_2	ACCCCTCCTCTCCCGATAG
HIF1A-AS3_RACE_3	ACAGCAATCAACAACACAGAAG
HIF1A-AS3_RACE_4	AAGCGATCCTTCTGCCTTGG

### Rapid amplification of cDNA ends (RACE)

To verify the correct sequence for the annotated terminal ends of HIF1A-AS3, we performed a 5’ and 3’ RACE. For 5’ RACE, we applied the Template Switching RT Enzyme Mix (New England Biolabs, Ipswich, MA, USA) in conjunction with HIF1A-AS3 specific primers and a template switching oligo (5’-TSO), following the manufacturer’s protocol. The 3’-RACE was conducted using an anchored 3’ oligo dT primer for cDNA synthesis as described above. After reverse transcription, cDNA was amplified by a nested PCR by means of Phusion Hot Start Flex 2X Master Mix (New England Biolabs, Ipswich, MA, USA). For the first and second PCR reaction, respectively, a TSO-specific outer or inner forward primer was used along with a corresponding HIF1A-AS3 specific reverse primer. The PCR products were cloned in the pMiniT vector (New England Biolabs, Ipswich, MA, USA) and sent to Eurofins Genomics Germany GmbH (Ebersberg, Germany) for sequencing. All primer and oligo sequences are shown in Table 1.

### Protein extraction and Western blot analysis

Cell lysis was conducted using the NucleoSpin TriPrep Kit according to manufacturer’s instructions. Protein concentration was measured by means of Protein Quantification Assay (Macherey-Nagel GmbH, Düren, Deutschland). Separation of proteins was achieved via sodium dodecyl sulfate–polyacrylamide gel electrophoresis (SDS-PAGE) applying precast stain-free Mini-PROTEAN^®^ gels. Activation by UV-light allowed direct protein visualization in the gel and after transfer to polyvinylidene difluoride (PVDF) membrane via Western blotting (both Bio-Rad Laboratories GmbH, Feldkirchen, Germany). For target protein detection, we used an antibody against Hif1α at 1:1000 dilution (Cell Signaling Technology Inc., Beverly, MA, USA) followed by StarBright Blue 700 Goat Anti-Rabbit at 1:40,000 dilution. Visualization and quantification was conducted by ChemiDoc MP imaging system and Image Lab software (both Bio-Rad Laboratories GmbH, Feldkirchen, Germany). According to the manufacturer (Bio-Rad Laboratories GmbH, Feldkirchen, Germany), a UV light mediated reaction allows the detection of tryptophan containing proteins on stain-free gels and western blot membranes after the transfer. Applying the ChemiDoc MP imaging system, transferred protein signals were detected and used for normalization to total protein in each lane. Here, we chose the automated image exposure time option. Automated detection of StarBright Blue 700 secondary antibody was achieved by a custom setup using a 596 nm filter.

### Cloning of OE vectors

Cloning was conducted according to standard procedures. Roughly, cDNA from HK-2 cells served as template for PCR based amplification of HIF1A, HIF1A-AS1, AS2, or AS3 using primers with overhangs, that carried cleavage sites for restriction endonucleases (Acc65I, HindIII, NheI, XbaI, or XhoI). Hot Start Taq 2X Master Mix or Phusion Hot Start Flex 2X Master Mix (all New England Biolabs, Ipswich, MA, USA) were used for PCR reaction. Depending on the polymerase used, genes were cloned using either a Topo TA Cloning Kit (ThermoFisher Scientific Inc., Waltham, MA, USA) or NEB^®^ PCR Cloning Kit vector Mix (New England Biolabs, Ipswich, MA, USA) and transformed into competent 10-Beta *E.coli*, which were supplied with the corresponding vector kit. Next, the TOPO/pMiniT plasmids and the expression vector pcDNA3.1(+) were cut by restriction endonucleases (listed above), ligated by T4 DNA ligase (New England Biolabs, Ipswich, MA, USA) and transformed into competent 10-Beta *E.coli*. Sequence identity was controlled by sequencing carried out by Eurofins Genomics Germany GmbH (Ebersberg, Germany).

### Graphic editing of data

SigmaPlot 14.0 was used to create graphs. With Inkscape 1.3.2, all figures were generated and edited. For reasons of clarity, this concerns a certain compressing of whole protein stain free western blot images and cropping of original immunoblotting images to exhibit only the detail of interest. When the details from two or more blots are shown in one figure, a white space separates each blot.

### Statistics

All statistical analyses were performed using SPSS Statistics Version 21. Normal distribution or homogeneity of variances were ascertained by Shapiro-Wilk and Levene’s test, respectively. To measure the overall significance of differences in more than two means, an analysis of variance (ANOVA) was conducted, before using Student’s t-tests for post hoc pairwise comparisons. Statistical difference was set at the 5% level of probability.

## Electronic supplementary material

Below is the link to the electronic supplementary material.

Supplementary Material 1

## Data Availability

All data generated or analyzed during this study are included in this published article.
