# Supplementary material for: Cell-type specific effects of the long non-coding RNA HIF1A-AS3 on HIF1A expression in kidney cells
Source: Sci Rep. 2025 Jul 30;15:27876. doi: 10.1038/s41598-025-12441-5 (PMC12310967; doi:10.1038/s41598-025-12441-5)
Supplement: Supplementary file 1 — Supplementary Material 1 [file 41598_2025_12441_MOESM1_ESM.pdf]

Supplementary data

The Hypoxia Inducible Antisense LncRNA HIF1A-AS3 Affects the Expression of Hif1□ and HIF1A-AS2 in HK-2 Cells but not Mesangial Cells

Simone Reichelt-Wurm*[1], Lena Knauss[1], Bettina Strasser[1], Kathrin Holler[1], Mona Scharf[1], Elke Eggenhofer[2], Markus Kretz[3], Bernhard Banas[1], Miriam C. Banas[1]

1) Department of Nephrology, University Hospital Regensburg, Regensburg, Germany
2) Department of Surgery, University Hospital Regensburg, Regensburg, Germany.
3) Institute for Molecular Medicine, MSH Medical School Hamburg, Hamburg, Germany

Supplementary Figure 1:

Expression of Hif1α protein under normoxia or hypoxia under normal and high glucose, respectively, or high mannitol

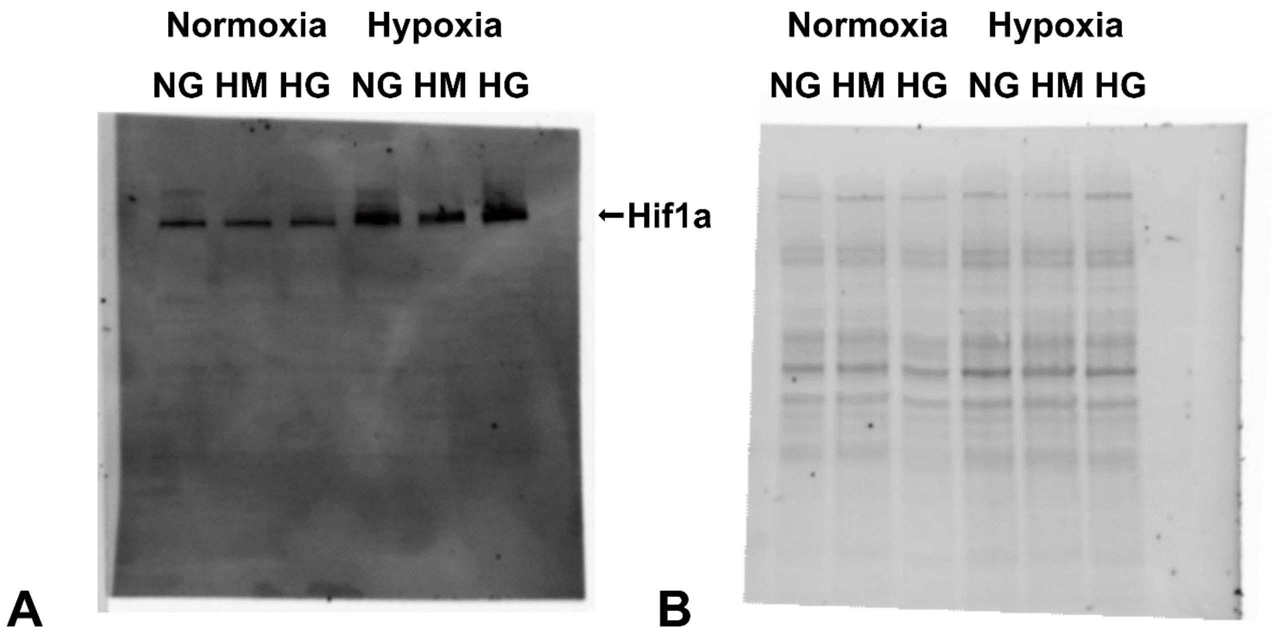

Suppl. Fig. 1: Expression of Hif1α protein in HK-2 cells under normal glucose (NG), high mannitol (HM), and high glucose (HG), respectively, under normoxia or hypoxia. (A) Detection of Hif1α by western blotting, (B) the corresponding whole protein stainfree blot image was used for normalization.

Supplementary Figure 2:

Expression of Hif1α protein after knockdown of HIF1A-AS1, AS2, and AS3 in HK-2 cells

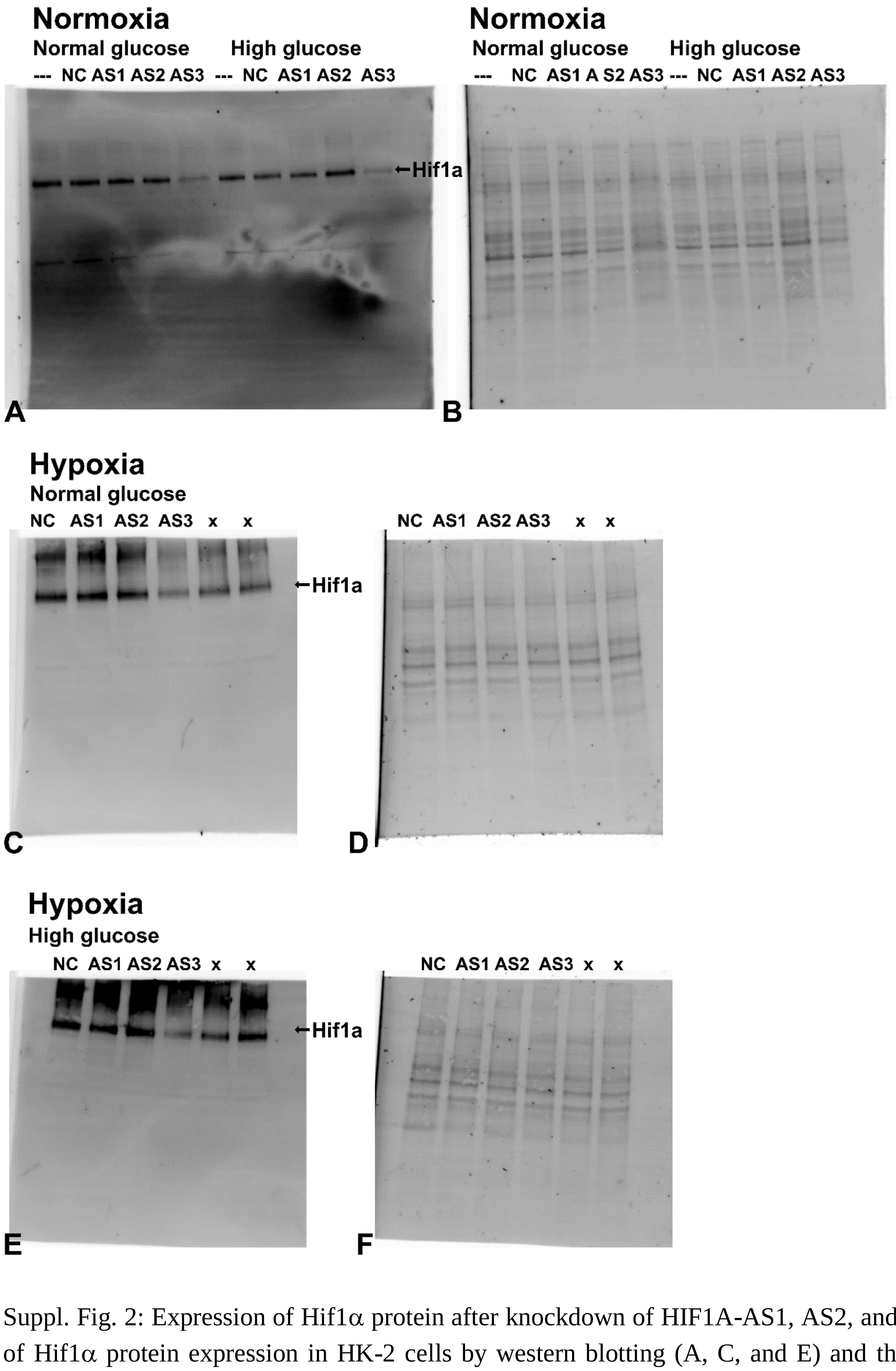

Suppl. Fig. 2: Expression of Hif1α protein after knockdown of HIF1A-AS1, AS2, and AS3. Detection of Hif1α protein expression in HK-2 cells by western blotting (A, C, and E) and the corresponding whole protein stainfree blot image used for normalization (B, D, and F) under normoxia and normal glucose (A and B), hypoxia and normal glucose (C and C), or hypoxia and high glucose (E and F) with simultaneous knockdown of HIF1A-AS1 (AS1), HIF1A-AS2 (AS2), and HIF1A-AS3 (AS3). Negative control is indicated by NC, --- represents no further treatment, and “x” corresponds to an additional treatment with is not subject of this work.

Supplementary Figure 3:

Effect of methods of hypoxia induction on the expression of HIF1A, HIF1A-AS2, and AS3

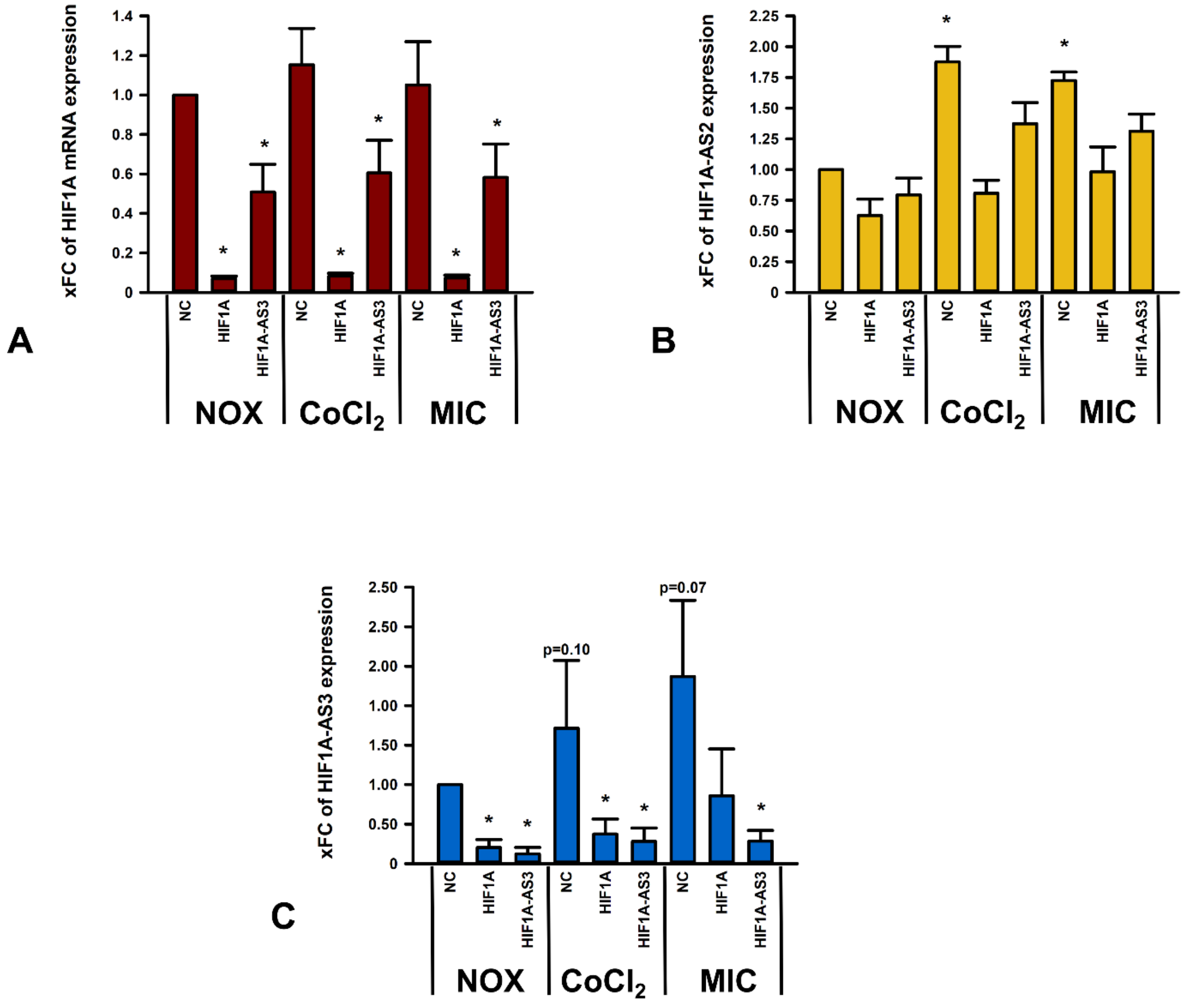

Suppl. Fig. 3: Expression of HIF1A, HIF1A-AS2 and AS3 after HIF1A or HIF1A-AS3 knockdown applying either $CoCl_2$ or a Modular Incubator Chamber (MIC) for hypoxia induction. Expression of HIF1A (A), HIF1A-AS2 (B), and HIF1A-AS3 (C) was examined after treatment with negative control (NC) siPools or siPools against HIF1A or HIF1A-AS3. Then, HK-2 cells were exposed to normoxia (NOX) or hypoxia caused by addition of $CoCl_2$ or culturing in a MIC (hypxic chamber). Bars + standard deviation (SD) are shown as x-fold change (xFC) using NG-normoxia samples as reference Significance was analyzed by ANOVA, followed by Student’s t-test. *) $p<0.05$; $n = 3$

Supplementary Figure 4:
Expression von HIF1A, HIF1A-AS1, AS2, and AS3 in RPTECs

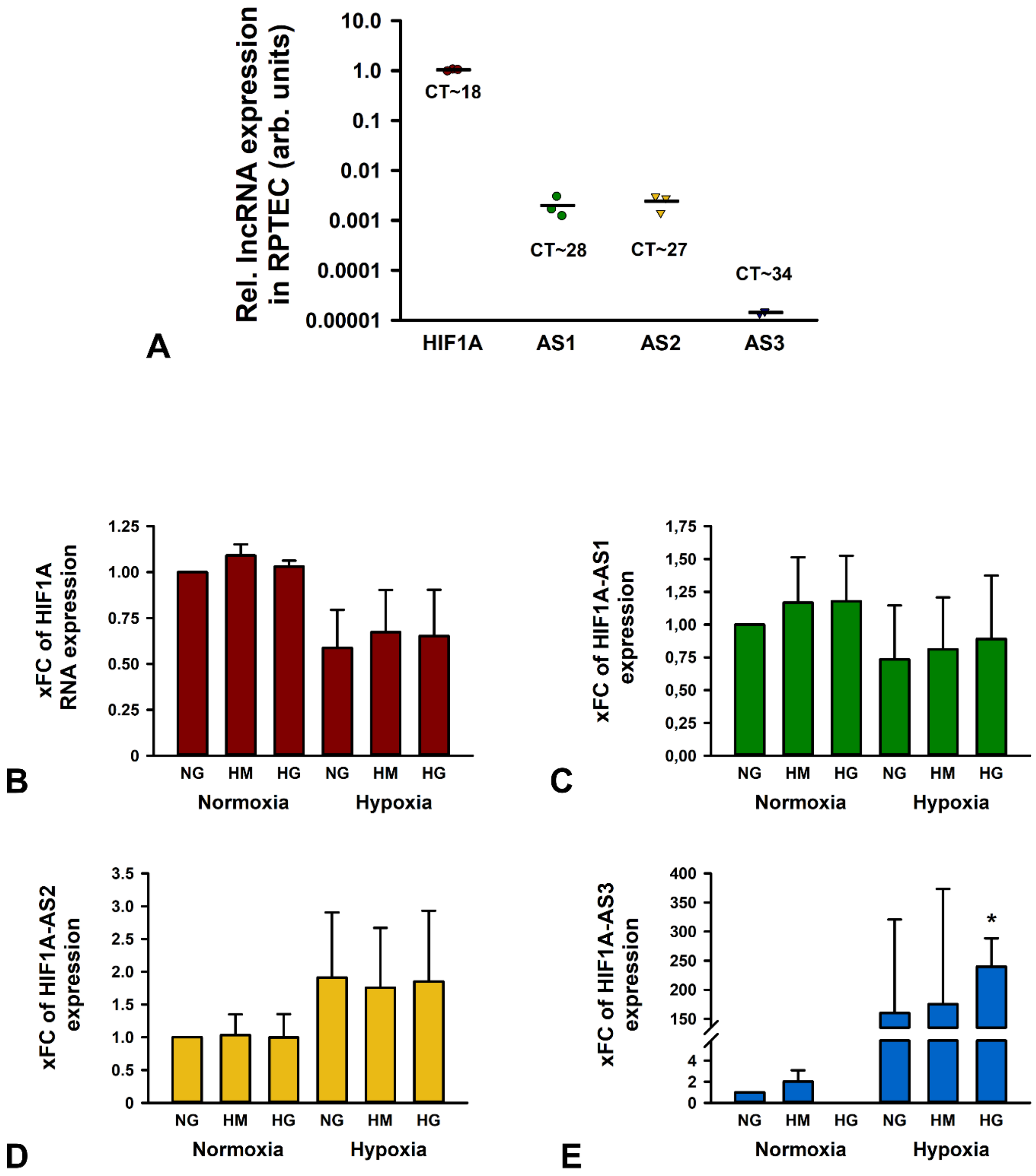

Suppl. Fig. 4: Expression of HIF1A and its AS lncRNAs. (A) RNA expression levels of HIF1A, HIF1A-AS1, AS2, or AS3 in untreated RPTECs determined by qPCR, depicted as logarithmic plot. Dots and triangles represents single results, bars the mean expression. CT: threshold cycle; n=3. Expression of HIF1A mRNA (B), HIF1A-AS1 (C), HIF1A-AS1 (D), and HIF1A-AS3 (E) lncRNA in RPTECs under normal glucose (NG), high mannitol (HM), and high glucose (HG), respectively, under normoxia or hypoxia. Bars + standard deviation (SD) are shown as x-fold change (xFC) using NG-normoxia samples as reference Significance was analyzed by ANOVA, followed by Student's t-test. *) $p<0.05$; $n = 3$

Supplementary Figure 5:
Expression of Hif1α protein after knockdown or overexpression of HIF1A-AS3 in hMCs

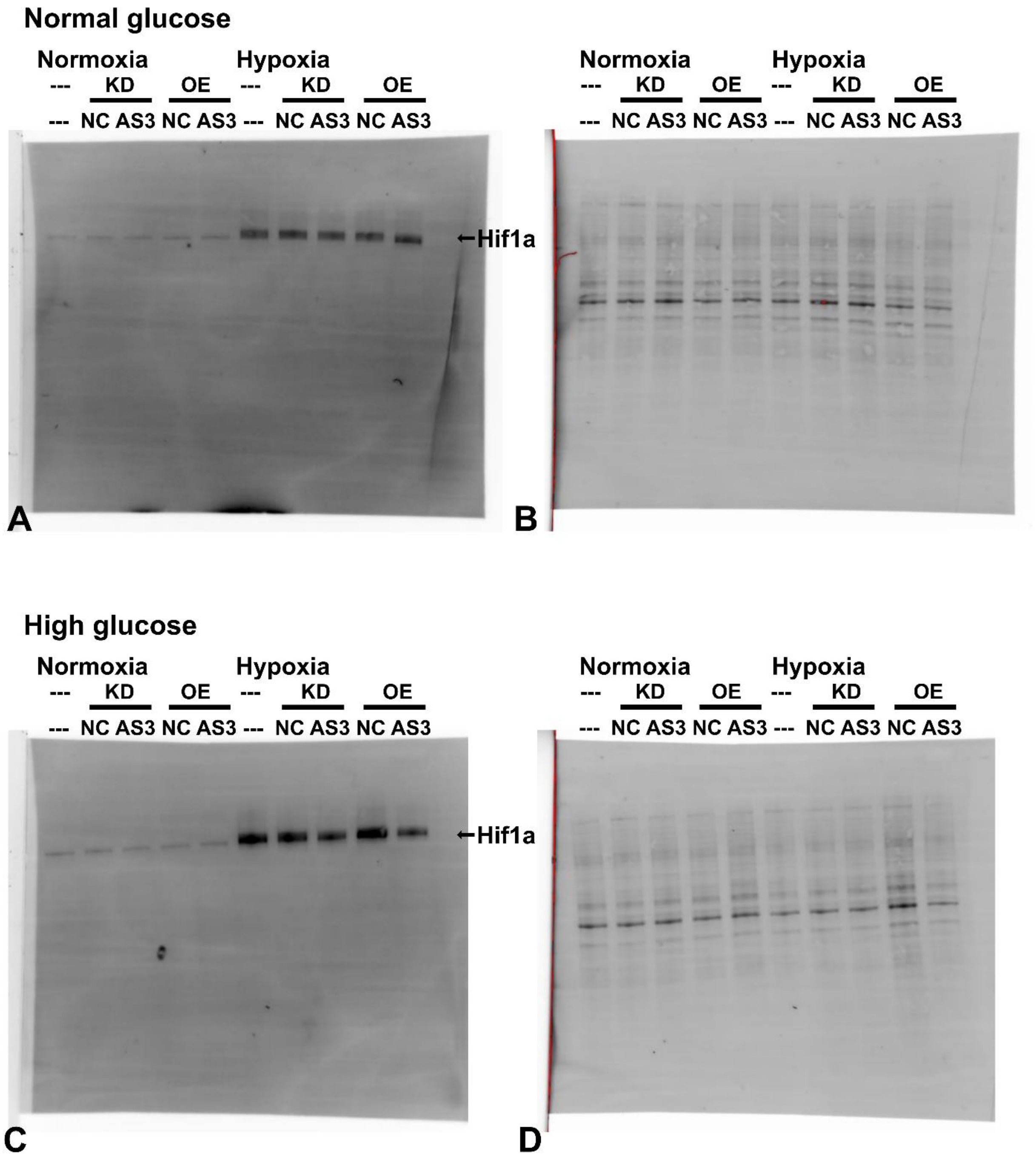

Suppl. Fig. 5: Expression of Hif1α protein after knockdown (KD) or overexpression (OE) of HIF1A-AS3. Detection of Hif1α protein expression in hMCs by western blotting (A and C) and the corresponding whole protein stainfree blot image used for normalization (B and D). HMCs were subjected to normoxia and hypoxia under normal glucose (A and B) and high glucose (C and D). Negative control is indicated by NC, --- represents no further treatment.
